# 5-year outcomes of salvage endoscopic nasopharyngectomy for recurrent nasopharyngeal carcinoma

**DOI:** 10.1186/s40463-020-00482-x

**Published:** 2021-02-17

**Authors:** Andrew Thamboo, Vishal S. Patel, Peter H. Hwang

**Affiliations:** 1 Division of Rhinology, Department of Otolaryngology – Head and Neck Surgery, Stanford University School of Medicine, Palo Alto, CA USA; 2 Department of Otolaryngology – Head and Neck Surgery, University of British Columbia, Vancouver, British Columbia Canada; 3 Department of Head and Neck Surgery, University of California of Los Angeles, Los Angeles, CA USA; 4 Department of Otolaryngology–Head and Neck Surgery, Stanford University School of Medicine, 801 Welch Road, Stanford, CA 94305 USA

**Keywords:** Recurrent nasopharyngeal carcinoma, rNPC, Endoscopic nasopharyngectomy, Long-term outcomes, 5-year outcomes, Overall survival, Disease-free survival, Tumor margin

## Abstract

**Objective:**

Recurrent nasopharyngeal carcinoma (rNPC) can be salvaged with re-irradiation, open nasopharyngectomy, and more recently endoscopic nasopharyngectomy. However, long-term outcomes of endoscopic approaches are lacking. Thus, we report 5-year outcomes following endoscopic nasopharyngectomy for rNPC.

**Methods:**

Patients who underwent endoscopic nasopharyngectomy for rNPC between January 2000 and January 2012 were retrospectively reviewed. Patients were included if they had their first endoscopic nasopharyngectomy at least 5 years prior to this study. Presenting (cTNM) status and recurrent (rTNM) status for each recurrence was determined. Outcomes included margin status, disease recurrence, death, and complication rates.

**Results:**

Thirteen patients were included. Four patients had a prior open nasopharyngectomy. Mean time follow-up was 74.3 months (range = 56.4–96 months). Negative margins were achieved in 77% of initial cases. Positive margins were associated with higher rT stages. Re-recurrence was seen in 6 patients, which was also associated with a higher cStage and rStage. All patients with positive margins had re-recurrence. Four patients required repeat endoscopic nasopharyngectomy and two received chemoradiation. All four with a second endoscopic procedure had further disease recurrence. Five-year local disease-free and overall survival rates were 53.9 and 84.6%, respectively. The minor complication rate was 52.6%, major operative complication rate was 0.0%, and late complication rate was 23.1%.

**Conclusion:**

Endoscopic nasopharyngectomy demonstrates promising 5-year overall survival rate for rT1 and rT2 cases of rNPC with favorable complication rates. Lower rStages were associated with a higher disease-free rate, and lower cStages were associated with improved overall prognosis. Close surveillance and prompt management of recurrences can be associated with favorable long-term tumor control.

**Level of evidence:**

4

**Graphical abstract:**

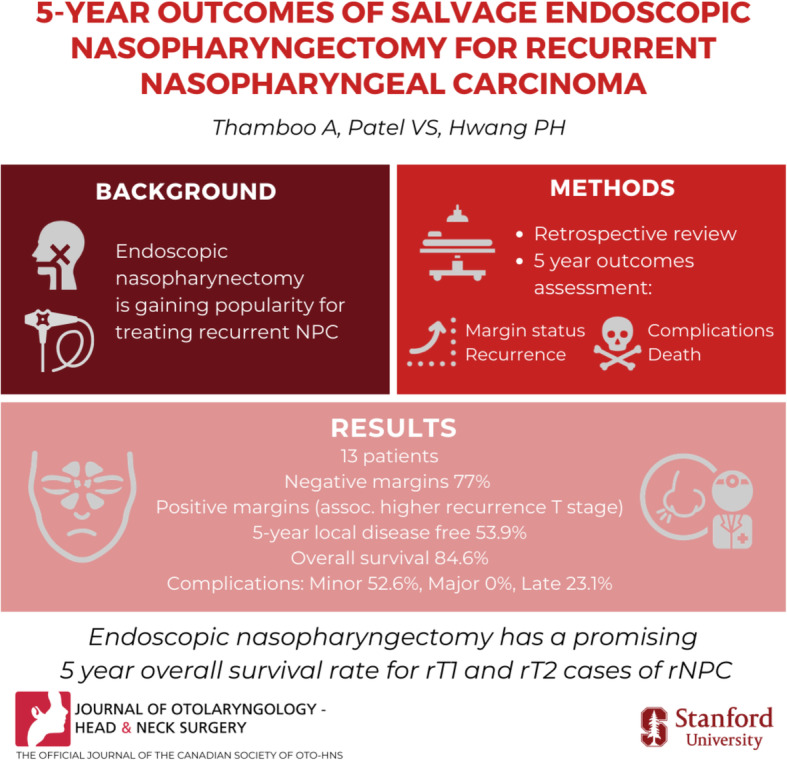

## Introduction

Surgical management of local, recurrent nasopharyngeal carcinoma (rNPC) has gained favor as a treatment alternative to re-irradiation. Traditional surgical options such as trans-palatal, trans-infratemporal fossa, trans-cervical, mid-face degloving, and maxillary swing approaches have been associated with 5-year overall survival (OS) ranging from 30 to 62%, favorable to reirradiation [1–3]. However such surgical techniques have been associated with a high degree of morbidity such as palatal defects, trismus, dysphagia, and nasal regurgitation [1, 3, 4].

A surgical approach that is gaining popularity for rNPC is the endoscopic nasopharyngectomy. With improved trans-nasal visualization provided by endoscopes, along with advances in endonasal instrumentation and a better understanding of endoscopic sinonasal anatomy, a number of sinonasal malignancies can now be managed endoscopically, including rNPC. For lower staged rNPC, this approach can potentially allow for similar outcomes as open nasopharyngectomy while limiting the morbidity associated with open surgical techniques. Based on a recent systematic review [5], the early survival outcomes of endoscopic nasopharyngectomy are quite favorable. There are six studies that report a 2-year OS of 59.4 to 100% [5–10], of which one was previously reported from our institution. However, given that endoscopic nasopharyngectomy is a relatively new approach, published long term outcomes are lacking, with only two studies from southern China and Malaysia that has reported 5-year OS of 78.1 and 50%, respectively [11, 12]. We now update our previously reported series with 5-year outcomes following endoscopic nasopharyngectomy for rNPC in North America.

## Methods

Approval was first obtained from the Stanford University Institutional Review Board (IRB). The Stanford Translational Research Integrated Database Environment (STRIDE), a standards-based informatics platform developed at Stanford that supports clinical and translational research, was utilized to collect a list of eligible patients [13]. We retrospectively reviewed all adult patients (≥ 18 years) with rNPC undergoing salvage endoscopic nasopharyngectomy at Stanford Medical Center from January 2000 to January 2012. Patients were included if they had their first endoscopic nasopharyngectomy at least 5 years prior to this study. The surgical approach is well described in our original study [6]. Patients with regional neck metastasis underwent concurrent neck dissection. Any additional adjuvant therapy following surgical management was also recorded. Post-operatively, patients underwent endoscopic surveillance every 1 to 2 months in the first 2 years and then every 3 to 6 months thereafter. Any suspicious lesions were biopsied. Patients also had yearly PET-CT or MRI scans to assess for local recurrence and distant metastasis.

In addition to the information obtained from the original study [6], to better characterize our cohort, the presenting TNM (cTNM) and recurrent TNM (r_n_TNM) status at each recurrence, along with their correlating presenting stage (cStage) and recurrent stage (r_n_Stage) were determined according to the 8th edition of the American Joint Committee on Cancer (AJCC) TNM classifications and stage groups. The World Health Organization (WHO) pathologic classification of NPC was also determined for each patient. Although most patients’ first recurrence after their initial radiation ± chemotherapy treatment was salvaged with an endoscopic nasopharyngectomy, a few patients presented to us with a 2nd recurrence after having had their first recurrence treated in the past with an open nasopharyngectomy. These patients who presented with recurrence after prior open nasopharyngectomy were offered an endoscopic procedure. As the goal of this study was to track disease recurrence after endoscopic surgery, we defined the recurrence status of patients prior to their first endoscopic nasopharyngectomy as r_0_TNM and r_0_Stage. Subsequent recurrence after the first endoscopic nasopharyngectomy was deemed the “first recurrence,” (r_1_TNM and r_1_Stage). Recurrence after a second nasopharyngectomy, if performed in applicable patients, was deemed the “second recurrence,” (r_2_TNM and r_2_Stage). All endoscopic procedures were performed with the intention to cure and improve overall survival. Surgery was offered to patients if the surgical team believed the tumor could be completely and safely resected.

Additional attention to the location of each recurrence and subsequent treatment (surgery and/or adjuvant therapy) are also reported. We report on the 5-year disease-free rate, overall-survival rate, and complications experienced by patients in this time frame. Minor complications were defined as crusting, epistaxis, purulence, pharyngitis, sinusitis, xerostomia, otitis media with effusion (OME), and eustachian tube dysfunction. Major operative complications were defined as carotid injury, cranial nerve injury, CSF leak, and velopharyngeal insufficiency. In addition, late complications due to comprehensive treatment were recorded including osteoradionecrosis (ORN), carotid rupture, temporal lobe necrosis, cranial neuropathy, dysphagia requiring a feeding tube, or trismus.

## Results

### Patient characteristics and presenting status

Twenty-five patients with recurrent, localized nasopharyngeal carcinoma were treated with endoscopic nasopharyngectomy; however only 13 patients met eligibility for 5-year follow-up. Patient characteristics are detailed in Table 1. Two patients had follow-up less than 5 years because of death prior to this time-point, but had their first endoscopic surgery at least 5 years prior to this study. Time of presenting diagnosis prior to their first endoscopic nasopharyngectomy averaged 50.5 months (range = 4.2–168 months). Initial radiation treatment for patients’ nasopharyngeal carcinoma averaged 68.6 Gy to the nasopharyngeal region and in 6 patients, an additional average dose of 57.6 Gy to the neck. Seven patients (54%) received adjuvant chemotherapy. Four patients had a prior open nasopharyngectomy before referred for an endoscopic procedure. Of these patients, timing of their open procedure averaged 76.2 months (range = 36.4–145 months) prior to their first endoscopic nasopharyngectomy.

**Table 1:** Patient characteristics

Patient #	Age at surgery / Gender	Ethnicity	cTNM	cStage	WHO class^d^	Time of diagnosis^a^	Initial treatment	Open procedure / Time^b^	Follow-up^c^ (months)
P1	61.9 / M	Caucasian	T2N2M0	Stage 3	III	24.4	60 Gy to nasopharynx	No	56.4
P2	50.3 / M	Taiwanese	T1N2M0	Stage 3	III	48.5	70 Gy to nasopharynx, 50 Gy to neck + chemo (5-FU, cisplatin)	No	57.2
P3	45.8 / M	Chinese	T1N0M0	Stage 1	II	36.2	IMRT (70 Gy to nasopharynx, 54 Gy to neck)	No	70.7
P4	60.4 / F	Japanese	T2N1M0	Stage 2	II	54.2	IMRT (70 Gy) + Chemo (cisplatin, 5-FU)	Yes / 36.7	70.5
P5	60.4 / M	African American	T1N0M0	Stage 1	III	5.2	IMRT (70 Gy to nasopharynx, 54 Gy to neck)	No	94.9
P6	80.8 / F	Chinese	T1N0M0	Stage 1	III	13.1	IMRT (70 Gy)	No	93.6
P7	34.9 / F	Taiwanese	T3N1M0	Stage 3	II	24.8	IMRT (70 Gy) + Chemo (cisplatin, 5-FU, bevacizumab)	No	89.1
P8	70.5 / M	Taiwanese	T3N0M0	Stage 3	III	168	External beam radiation	Yes / 86.8	96
P9	63.8 / M	Filipino	T1N1M0	Stage 2	III	24.0	IMRT (66.6 Gy to nasopharynx, 59.4 Gy to neck) + Chemo (cisplatin)	No	86
P10	69.9 / M	Filipino	T1N1M0	Stage 2	II	13.0	IMRT (70 Gy to nasopharynx, 56.4 Gy to neck)	No	67.2
P11	42.5 / M	Chinese	T1N3M0	Stage 4a	III	84.7	70 Gy to nasopharynx, 72 Gy to neck + Chemo (5-FU, cisplatin)	Yes / 36.4	60.6
P12	45.5 / M	Chinese	T1N2M0	Stage 3	III	4.2	IMRT (70 Gy) + Chemo (5-FU, cisplatin)	No	82.2
P13	44.6 / F	Chinese	T1N2M0	Stage 3	I	156	Radiation + Chemo	Yes / 145	78.2

^a^“Time of diagnosis” of the presenting nasopharyngeal carcinoma is denoted by the number of months the diagnosis was made prior to the first endoscopic nasopharyngectomy

^b^“Time” of open nasopharyngectomy is denoted by the number of months the procedure was performed prior to the first endoscopic nasopharyngectomy

^c^“Follow-up” is calculated as the number of months of patient follow-up after the first endoscopic nasopharyngectomy

^d^Tumor classifications: I = keratinizing, II = non-keratinizing, III = undifferentiated, non-keratinizing, associated with EBV

### Recurrence status (r_0_) prior to endoscopic nasopharyngectomy

Recurrent TNM status and correlating r_0_Stages prior to endoscopic nasopharyngectomy are described in Table 2. The majority of patients (61.5%) were r_0_T1. Among the 13 initial endoscopic nasopharyngectomies, negative margins were seen in 10 patients (77%). Positive margins were seen in patients P1, P4, and P10 at the fossa of Rosenmuller, parapharyngeal space, and oropharynx, respectively. The r_0_T status for each of these patients were r_0_T2, r_0_T2, and r_0_T1, respectively. Adjunctive radiation boosts were performed via CyberKnife SRT (12 Gy) for patients P1 and P4 and via IMRT (60 Gy) for P10 (for positive margins). In addition brachytherapy was delivered for P12 as decided by tumor board prior to surgery.

**Table 2:** Recurrence status (r_0_) prior to endoscopic nasopharyngectomy

Patient #	r_**0**_TNM	r_**0**_Stage	Location of recurrence	Surgical margins	Adjuvant therapy
P1	T2N1M0	Stage 2	Nasopharynx, parapharyngeal space	+ (fossa of Rosenmuller)	CyberKnife SRT (12Gy) + Chemo (docetaxel, cisplatin, 5-FU)
P2	T1N0M0	Stage 1	Nasopharynx	–	None
P3	T1N0M0	Stage 1	Nasopharynx, nasal cavity	–	None
P4	T2N0M0	Stage 2	Nasopharynx, parapharyngeal space	+ (parapharyngeal space)	CyberKnife SRT (12Gy)
P5	T1N0M0	Stage 1	Nasopharynx	–	None
P6	T1N0M0	Stage 1	Nasopharynx, nasal cavity	–	None
P7	T1N0M0	Stage 1	Nasopharynx	–	None
P8	T3N0M0	Stage 3	Nasopharynx, sphenoid sinus	–	None
P9	T1N0M0	Stage 1	Nasopharynx	–	None
P10	T1N1M0	Stage 2	Nasopharynx, oropharynx	+ (oropharynx)	IMRT (60 Gy) + Chemo (cetuximab)
P11	T3N0M0	Stage 3	Nasopharynx, floor of sphenoid sinus	–	None
P12	T1N0M0	Stage 1	Nasopharynx	–	Brachytherapy
P13	T1N0M0	Stage 1	Nasopharynx	–	None

### First recurrence (r_1_) after endoscopic nasopharyngectomy

Six patients (P1, P2, P4, P8, P10, P11) had a recurrence despite initial endoscopic nasopharyngectomy. All six patients had a cStage of 2 or greater and five (83%) had an r_0_Stage of 2 or greater. However, in regards to local T staging, only 1 (17%) had cT status of T3 or greater and 2 (33%) had a r_0_T of T3 or greater. Also, of all patients with r_0_Stage 2 and r_0_Stage 3, 100% had a second recurrence. All three patients with previous positive margins also had a second recurrence, despite adjuvant radiation therapy.

First recurrence TNM (r_1_TNM) status and correlating r_1_Stage were defined, as seen in Table 3. Four (P1, P2, P8, P11) were managed with a second endoscopic nasopharyngectomy and three had negative margins. P8 had positive margins at the infratemporal fossa and was given a CyberKnife SRT (1.6 Gy) boost to the area of positive margin. Two patients (P4, P10) had parapharyngeal involvement with extension encasing the carotid arteries and were thus deemed non-surgical and underwent systemic chemotherapy with combinations of docetaxel, cisplatin, or cetuximab. After medical treatment, P4 remains high functioning with stable disease; however, P10 is alive but in comfort care, requiring a tracheosotomy and feeding tube.

**Table 3:** First recurrence (r1) after endoscopic nasopharyngectomy

Patient #	Location of first recurrence	r_**1**_TNM / r_**1**_Stage	Time to second recurrence (months)	Subsequent treatment	Surgical margins	Adjunctive therapy
P1	Nasopharynx, posterior inferior turbinate	T1N0M0 / Stage 1	24.6	Endoscopic nasopharyngectomy, partial medial maxillectomy	–	none
P2	Fossa of Rossenmuller	T1N0M0 / Stage 1	5.6	Endoscopic nasopharyngectomy	–	none
P4	Parapharyngeal space (carotid extension)	T2N0M0 / Stage 2	34.5	Chemo via docetaxel and cisplatin (currently alive with stable disease)	N/A	N/A
P8	Nasopharynx; pterygomaxillary fossa; infratemporal fossa	T4N0M0 / Stage 4	25.2	Endoscopic nasopharyngectomy, dissection of pterygomaxillary fossa, dissection of infratemporal fossa	+ (infratemporal fossa)	CyberKnife SRT (1.6Gy)
P10	Parapharyngeal space (carotid extension), skullbase, bilateral retropharyngeal nodes	T3N2M0 / Stage 3	14.4	Chemo via docetaxel and cetuximab (currently alive in comfort care)	N/A	N/A
P11	Sphenoid sinus; pterygopalatine fossa	T3N0M0 / Stage 3	9	Sphenoidotomy; pterygopalatine fossa dissection	–	None

### Second recurrence (r_2_) after endoscopic nasopharyngectomy

A second recurrence was seen in all four patients (P1, P2, P8, P11) who underwent a second endoscopic nasopharyngectomy (Table 4). Of the four patients, 75% of them were r_0_Stage 2 or greater, and 50% were r_1_Stage 3 or greater. Their initial presenting stage was cStage 3 or 4. In regards to local T staging, 50% had r_0_T of T3 or greater, and 50% had r_1_T of T3 or greater. These patients also had their TNM status and correlating stage updated for their second recurrence: three patients were r_2_Stage 3 and two were r_2_Stage 4. P1 had extensive nodal disease and was subsequently treated with systemic chemotherapy. P2 had metastasis to the right tonsil and hard palate, confirmed by biopsy, and subsequently treated with systemic chemotherapy. Shortly after, P2 had further disease progression with intracranial extension and was given palliative chemotherapy. P8 had recurrence extending into the dura and received palliative chemotherapy. P11 had isolated left sphenoid sinus recurrence and was treated with a limited endoscopic nasopharyngectomy and left sphenoidotomy with negative margins. However, shortly after, P11 had metastasis to the lungs and received palliative chemotherapy.

**Table 4:** Second recurrence (r_2_) after endoscopic nasopharyngectomy

Patient #	Time to recc. (months)	Location of second recurrence	Subsequent treatment	r_**2**_TNM/ r_**2**_Stage	Surgical margins	Adjunctive treatment	Disease progression	Current status	Time to death (years)
P1	3.6	Nasopharynx, retropharyngeal, suboccipital, submental nodes	Chemotherapy	T1N2M0 / Stage 3	N/A	N/A	N/A	Dead	4.7
P2	5.7	Nasopharynx, tonsil, hard palate	Endoscopic nasopharynx biopsy, right tonsillectomy	T4N0M1 / Stage 4	+	Chemotherapy	Intracranial spread	Dead	4.7
P8	33.6	Nasopharynx w/ extension into dura	Palliative chemotherapy	T4N0M0 / Stage 4	N/A	N/A	N/A	Alive with disease	N/A
P11	16.3	Sphenoid sinus	Endoscopic nasopharyngectomy, sphenoidotomy	T3N0M0 / Stage 3	–	None	Metastasis to lungs	Dead	5.6

### Local control, disease free and overall survival rates

**Fig. 1:**
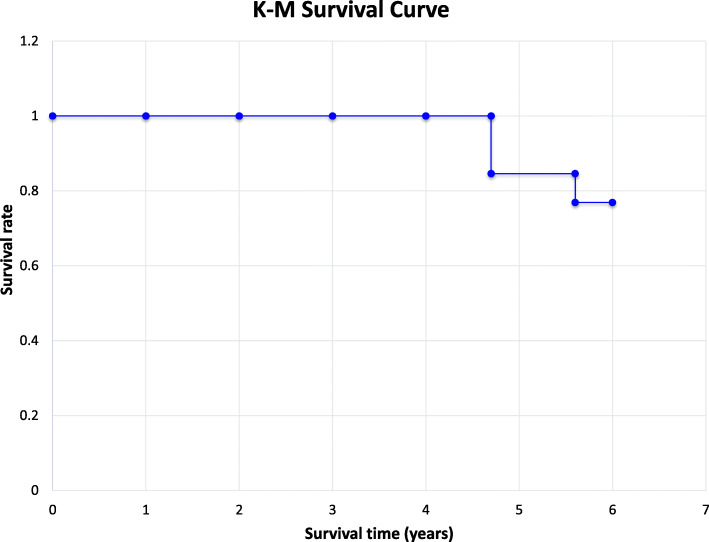
The Kaplan-Meier survival curve for patients undergoing salvage endoscopic nasopharyngectomy for recurrent NPC is illustrated. Two patients (P1 & P2) died at 4.7 years, just prior to the 5-year mark, with an additional death (P11) shortly after at 5.6 years

Local control of tumor was achieved in eight patients (P3, P5, P6, P7, P9, P11, P12, P13) after their first endoscopic nasopharyngectomy to their latest follow-up for a 5-year local control rate of 61.5% (Fig. 1). Seven of these patients remain disease-free for a 5-year disease free rate of 53.9%. Although one patient (P11) achieved local control of tumor, he developed distant metastases to the lungs. Of the disease-free cohort, 3 were cStage 1 (43%), 1 was cStage 2 (14%), and 3 were cStage 3 (43%). In regards to their r_0_Stage, all 7 were r_0_Stage 1 (100%).

**Fig. 2:**
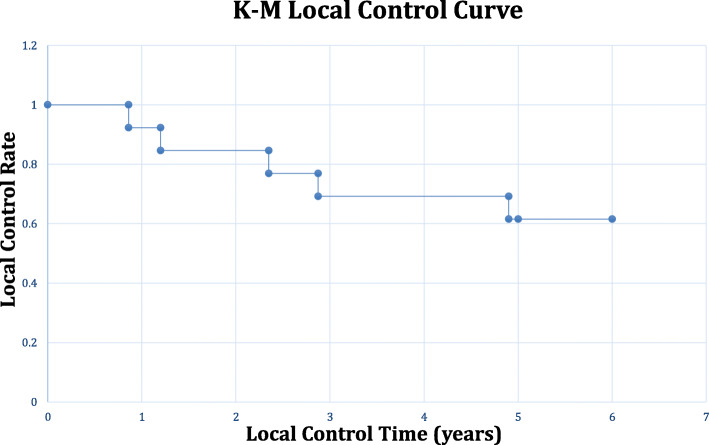
The Kaplan-Meier local tumor control curve for patients undergoing salvage endoscopic nasopharyngectomy for recurrent NPC is illustrated. Local control is defined as no evidence of disease after resection or if positive margins, no evidence of disease or tumor size change after adjuvant therapy. Five patients underwent local disease progression for a 5-year local control rate of 61.5%

In addition to the seven patients described above, six patients had disease (P1, P2, P4, P8, P10, P11) of which a total of three patients (P1, P2, P11) died due to disease progression (Fig. 2). Two of these patients (P1, P2) died 4.7 years after their initial endoscopic nasopharyngectomy. P11 died 5.6 years after their initial endoscopic and thus was not included in the 5-year rate. Of the patients that died, all were cStage 3 or cStage 4. Therefore, for the entire cohort, the 5-year OS was 84.6%.

The 5-year disease free and 5-year OS rates were also analyzed based on whether the stage at initial surgery or at recurrence was early or advanced. For patients with early cStages [1, 2], the disease-free rate was 67% and overall survival rate was 100%. For patients with early r_0_Stages [1, 2], the disease-free rate was 64% and overall survival rate was 82%. On the contrary, for advanced cStages [3, 4], the disease-free rate was 43% and the overall survival rate was 71%. For advanced r_0_Stages [3, 4], the disease-free rate was 0% and overall survival rate was 50%.

### Complications

There were no additional operative complications experienced by our patients after the 2-year follow-up as previously reported in our original study [6]. All patients had some degree of post-operative crusting that resolved within a month with debridements and saline rinses. The most common side effects were otitis media with effusion (OME) seen in 4 (30.8%) cases. Purulence was seen in three (23.1%) patients, which was adequately treated with antibiotics. Mild epistaxis occurred after one (7.7%) procedure and was controlled with silver nitrate application. There were no major operative complications. The total minor complication rate was 52.6% and the major complication rate was 0.0%.

Among late complications secondary to comprehensive treatment, osteoradionecrosis developed in three (23.1%) patients. One case of ORN involving the external auditory canal occurred in P2, eight months after his second endoscopic nasopharyngectomy. The second case of ORN involving the posterior inferior turbinate occurred in P5, 7 months after his initial endoscopic procedure. The third case of ORN involving the skull base and sphenoid sinus occurred in P11, one year after his third endoscopic procedure. All cases were managed with saline irrigations, routine debridement, and antibiotics if secondary infection developed. One new case of trismus (7.7%) developed in P11. Temporal lobe necrosis occurred in P2 (7.7%), although no cognitive deficits were seen. Carotid rupture occurred secondary to progressive skull base ORN in P11. Other than P2, P5, and P11, all other patients experienced no late complications. The total late complication rate was thus 23.1%.

## Discussion

In the last decade with further familiarity and advancement of endoscopic techniques, the endoscopic nasopharyngectomy has been increasingly utilized for local recurrences of NPC, providing potentially fewer complications compared to open surgery or reirradiation. However, this endoscopic approach is still relatively new with limited information and experience in North America [14]. Due to the location of our institution on the west coast of North America, the majority of patients seen at our center are first- or second-generation immigrants from Asia. Though the majority of current studies regarding endoscopic nasopharyngectomy report favorable 2-year OS rates, no studies from North America report on 5-year OS in order to properly establish this approach.

Endoscopic nasopharyngectomy shows favorable 5-year overall survival rates. The previous study on 5-year outcomes reports a 78% OS, and we report an 84.6% 5-year OS. Similarly, our surgical cohort contained patients primarily with rT1–2 staged rNPC, with 61.5% being rT1. We did have two patients with rT3 but they were staged higher because of sphenoid sinus involvement. Comparing these findings to those for similarly staged rT1–2 tumors treated by IMRT (mean 5-year OS = 71%, range = 58–80% [15–17]) and open surgical techniques (mean 5-year OS = 56%, range = 47–65% [1, 2]) may suggest that patients with low rT staged rNPC treated by endoscopic nasopharyngectomy have comparable 5-year OS to reirradiation with IMRT and open procedures [18, 19]. However, it is important to acknowledge that inherent selection bias exists in selecting patients for an open versus endoscopic nasopharyngectomy that may not be reflected in the rT staging alone. In addition, a study by You et al. [20], performed a case-control study to determine if endoscopic nasopharyngectomy was beneficial compared to IMRT in patients with similarly staged rNPC. The study by You et al. showed that endoscopic nasopharyngectomy is more effective in maximizing survival and quality of life benefits while minimizing treatment related complications and medical costs compared to IMRT. Given this, and the survival outcomes demonstrated by this study, appropriately chosen rT1-T2 staged rNPC should be considered for endoscopic surgical dissection versus reirradiation. Patients with rT3 staged rNPC due to sphenoid sinus involvement may also be feasibly treated with an endoscopic approach, however more data is required to assess how these patients do in the long-term.

Historically, the American Joint Committee on Cancer (AJCC) rTNM staging system has been used to guide both treatment and prognosis with rT1–2 tumors often treated surgically and rT3–4 tumors treated with chemoradiation. However, it is becoming evident that the staging system does not encompass the evolving surgical techniques of our current generation. You et al. suggested a revised non-validated TNM staging system for rNPC based on surgical resectability that may better stratify patients as surgical versus IMRT candidates [21]. They proposed some rT3 cancers with current surgical technique and tools can be managed surgically. Ultimately, the resectability of a tumor should drive management, which is imperfectly defined by the TNM staging system. While previously only open nasopharyngectomies could allow access to areas such as the orbit (T4) and masticator space (T4), the endoscope can also reach certain T3 and T4 tumors, such as those with extension into the sphenoid sinus, the infratemporal fossa, and the orbit. Thus, T staging alone also does not dictate when an open versus endoscopic approach would be appropriate. In cases where the endoscope can achieve the same gross total resection as open, we advocate that oncological teams consider the endoscopic approach, in lieu of open approaches, which may be associated with morbidities such as cosmetic scars, palatal defect, nasal regurgitation, and trismus [1]. In our endoscopic case-series, we found no major perioperative complications and only minimal, transient complications such as crusting and OME. Other groups reporting on endoscopic nasopharyngectomy also report similar findings [5, 8, 22, 23].

TNM staging has also been used to help guide patient prognosis, and historically the presenting tumor stage has best predicted overall survival for head and neck cancers. In our study, a total of two patients with cStage 3 died due to disease progression before 5-year follow-up and one patient with cStage 4 succumbed to his disease just after the 5-year mark. For patients with presenting early (cStages 1–2), the disease-free rate was 67% and overall survival rate was 100%, whereas advanced cStages [3, 4], the disease-free rate was 43% and the overall survival rate was 57%. However, it is important to note, four other patients with cStage 3 did well over their 5-year surveillance without evidence of disease suggesting that advanced cStages did not necessarily predict increased mortality. As for the rStage, the three patients that died presented with varying stages, r_0_Stage 2, r_0_Stage 1 and r_0_Stage 3 respectively, and it is also difficult to conclude from this data whether the rStage plays a significant predictive role in patients’ overall survival. However, in regards to disease status, r_0_Stage may be more predictive than cStage. Of patients with known disease at 5-year follow up, 83% were r_0_Stage 2 or greater and of patients that were disease free, 100% were r_0_Stage 1.

Ultimately, it is important to acknowledge that the presenting or recurrent staging may not be the only important prognostic factors. Histological subtypes are independent prognostic factors, with non-keratinizing tumors having improved outcomes compared to keratinzing tumors [24, 25]. EBV positivity, which is associated with non-keratinizing carcinomas, is also associated with improved OS [26]. These findings may be due to the fact that non-keratinizing tumors are more radiosensitive than keratinizing types [27]. Consequently, our study did not include histologic subtypes, and it is difficult to conclude if this factor played a prognostic role in the outcomes of our salvage nasopharyngectomies.

**Fig. 3:**
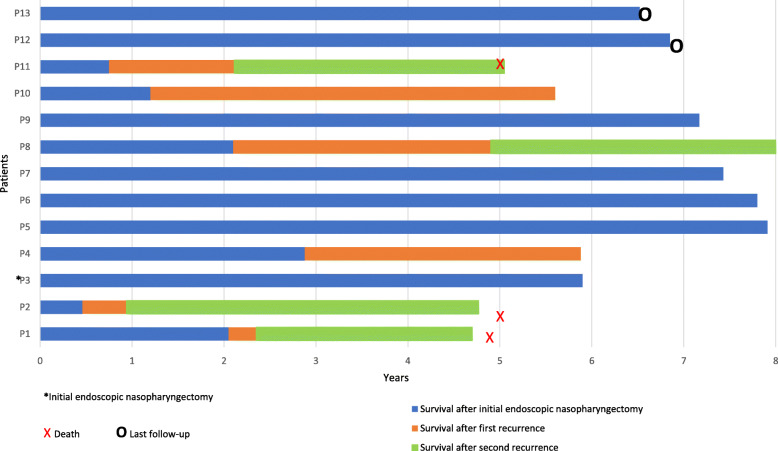
The number of recurrences, timing to each subsequent recurrence, and overall survival time after initial endoscopic nasopharyngectomy for each patient is illustrated

This study also shows that among those who recur following salvage endoscopic nasopharyngectomy, local and regional control can still be achieved if patients receive close endoscopic surveillance with prompt initiation of re-resection or adjuvant therapies for recurrence. In our series, patients underwent endoscopic surveillance every 1 to 2 months in the first 2 years after initial radiation therapy and then every 3 to 6 months thereafter. Patients also had yearly PET-CT or MRI scans to assess for occult local recurrences and distant metastasis with a very low threshold to biopsy suspicious lesions. In our cohort, subsequent endoscopic nasopharyngectomies were offered for patients with recurrent, local disease in which complete surgical resection was both safe and possible. Of the 6 recurrences seen, 4 were deemed safely resectable and negative margins were achieved in 3 of 4 (75%) cases. Despite the high rate of negative margins in these recurrent cases and adjuvant chemo-radiation therapies, all 4 patients experienced disease progression with death in 3 cases, likely representing a more aggressive, treatment resistant tumor nature. Although these repeat resections did not prevent ultimate mortality in these patients, two died just prior to 5 years (both at 4.7 years) and one died after 5 years of follow-up. This finding may suggest value in repeat operations by helping limit local disease progression and thus extending overall survival. Close surveillance plus prompt management with a combination of surgery, radiation, or chemotherapy can allow for satisfactory 5-year local control of disease (61.5%) and overall survival rates (84.6%) (Figs. 1, 2 and 3).

As this study includes the same patient cohort as our previous publication on 2-year outcomes after endoscopic nasopharyngectomy, we were also able to discern longer-term trends in disease progression not reflected in the 2-year study alone. Our cohort suggests that patients without recurrence in the first 2 years after endoscopic nasopharyngectomy are more likely to remain disease free at 5 years. Patients who recur within 2 years despite negative margins at initial endoscopic nasopharyngectomy are more likely to continue to recur again despite re-resection, with disease progression and/or death by 5 years. Patients with positive margins at initial surgery (associated with higher r_0_T stages) are likely to experience disease progression and/or death over 5 years despite adjuvant chemoradiation.

It is important to acknowledge some limitations of our study. First, our study design is a retrospective case series and direct comparison to other surgical techniques or reirradiation was not assessed. Additionally, the disease characteristics of our cohort were heterogeneous. Some had open surgery prior to receiving their endoscopic nasopharyngectomy, while others had chemoradiation only; furthermore, presenting and recurrent tumor stages for each patient, as well as pathologic classifications were variable. However, despite these limitations, this study achieves its goal by adding to the much lacking literature on 5-year outcomes of endoscopic nasopharyngectomy.

## Conclusions

Endoscopic nasopharyngectomy shows an excellent 5-year overall survival rate of 84.6% for rT1 and rT2 cases of rNPC with a favorable complication rate. Lower rStages were associated with a higher disease-free rate, and early cStages were associated with improved overall prognosis. Patients who are candidates for endoscopic nasopharyngectomy have by nature a more treatment-resistant tumor that may recur multiple times within the first 5 years after surgery. Nevertheless, close surveillance and identification/resection of recurrences at an early stage can be associated with favorable long-term control of tumor.

## Data Availability

The datasets during and/or analysed during the current study available from the corresponding author on reasonable request.
